# Unsupervised Driving Behavior Primitive Inference via Hierarchical Segmentation and Context-Aware Clustering

**DOI:** 10.3390/s26092744

**Published:** 2026-04-29

**Authors:** Lu Zhang, Tao Li, Xuelian Zheng, Wenyu Kang, Yuhan Fu

**Affiliations:** 1 China Automotive Technology and Research Center Co., Ltd., Tianjin 300300, China; litao2017@catarc.ac.cn; 2 State Key Laboratory of Engines, Tianjin University, Tianjin 300072, China; 3 Transportation College, Jilin University, Changchun 130012, China; zhengxuelian@jlu.edu.cn (X.Z.);

**Keywords:** driving behavior primitive, multi-dimensional time series, sequence segmentation, segment clustering, semantic analysis

## Abstract

Driving behavior primitives serve as fundamental building blocks for modeling and semantically interpreting time-series driving behavior. Extracting behavior primitives is challenging due to the high dimensionality and complex interdependencies among behavioral variables, as well as the rich temporal dynamics of real-world driving maneuvers. This paper proposes an unsupervised two-stage framework that optimizes time-series segmentation and segment clustering to yield interpretable and context-aware behavior primitives. First, a Hierarchical Bayesian Model-based Agglomerative Sequence Segmentation (H-BMASS) method is introduced that decouples longitudinal and lateral driving behaviors and performs hierarchical segmentation. This design mitigates under-segmentation by ensuring that change points reflect genuine behavioral transitions. Second, to cluster driving segments of varying durations into a finite set of primitive types, an Integrating Numerical and Trend Discretization Latent Dirichlet Allocation (INT-LDA) model is developed. The model combines variables’ temporal trend discretization with numerical discretization to create symbolic representations of driving data, thereby preserving the essential time dependency of driving behavior and improving segment clustering accuracy. Evaluated on naturalistic driving data collected from a high-fidelity simulator, the proposed framework identifies five distinct behavior primitives with clear physical interpretations. The resulting primitives provide a compact, semantically rich representation of driving behavior, facilitating driver modeling, decision prediction, and scenario-based testing for autonomous vehicles.

## 1. Introduction

Driving behavior encompasses the decisions and reactions exhibited by drivers in response to dynamic traffic environments, reflecting their distinct behavioral patterns. These characteristics are instrumental in a wide range of applications, including driving style analysis and classification [1,2,3], behavior modeling [4,5], driver behavior assessment and risk prediction [6,7,8,9], complex interaction understanding between multi-vehicles [10], trajectory prediction and decision-making for AVs [11,12,13,14], and so on. Consequently, gaining deep insights into driving behaviors is pivotal for elucidating driving habits, improving traffic safety, and advancing the development of autonomous driving technologies [15,16].

Traditionally, research on driving behavior has primarily focused on macroscopic description, explanatory analysis, and predictive modeling. A core tenet of these studies is the treatment of driving as a continuous and homogeneous process [17,18], aiming to extract population-level commonalities or individual differences from large-scale datasets. In the realm of driving style analysis, scholars typically employ statistical analysis and regression models to construct behavioral features. These features are subsequently processed using techniques such as Principal Component Analysis (PCA) and clustering algorithms to identify shared patterns and distinctions, thereby enabling effective driver classification [19,20,21,22,23]. Regarding explanatory and predictive modeling, existing studies predominantly utilize physical mechanism-based models or neural network architectures. The primary objective of these approaches is to accurately reproduce observed driving data, striving for optimal fitting performance on historical datasets [24,25,26].

However, a fundamental discrepancy exists between traditional modeling assumptions and actual human driving cognition. Drivers do not consciously manage vehicle control as a continuous, micro-level, time-varying execution process. Instead, they typically achieve their goals through a combination of simple, high-level actions that reflect strategic decision-making in complex situations [27,28]. Experienced drivers develop subconscious “muscle memory” patterns; when encountering diverse traffic environments, they instinctively retrieve and apply specific behavioral modes without focusing on the microscopic details of operational execution.

Consequently, to gain deeper insights into this underlying decision-making process, it is essential to extract the fundamental building blocks that encapsulate distinct, interpretable actions from the complex continuum of driving maneuvers. Here, driving maneuvers are defined as macroscopic, goal-oriented operations sustained over extended durations, such as lane changing, car-following, and turning. These fundamental units, referred to as driving behavior primitives [29,30,31,32], aim to decompose intricate maneuvers and scenarios into smaller, manageable, and semantically meaningful segments. This decomposition facilitates fine-grained behavior analysis and enhances the robustness of decision-making models. Serving as a foundational framework for understanding, modeling, and predicting driver actions, these primitives are critical for the advancement of Intelligent Transportation Systems (ITS), Advanced Driver-Assistance Systems (ADAS), and Autonomous Vehicles (AVs) [33,34].

Driving behavior sequences, typically represented as multi-dimensional time-series data, are inherently continuous and variable. The segmentation process divides this continuous data stream into distinct episodes, or segments, that correspond to specific driving actions or states. This process is predominantly achieved through data-driven approaches leveraging naturalistic driving data. A widely adopted strategy involves Bayesian Nonparametric Learning (BNPL) techniques, such as those based on Hidden Semi-Markov Models (HSMMs) and Hierarchical Dirichlet Processes (HDPs). These methods possess the capability to automatically discover latent patterns within multi-dimensional time-series data without requiring prior knowledge of the number of states [5,15,16,27,35,36,37,38,39]. Consequently, they facilitate the identification of primitives representing distinct driving patterns, including accelerating, braking, turning, lane-keeping, and lane-changing.

Once the continuous sequence is segmented into discrete episodes, the subsequent step involves clustering these segments to identify recurring patterns, which are formally defined as driving behavior primitives [40]. The goal of clustering is to group similar segments, thereby abstracting raw low-level data into higher-level semantic actions. Existing methodologies can be categorized into hard clustering and soft clustering.

Due to the variability in segment durations, hard clustering algorithms typically require a preprocessing step to extract static features to unify dimensionality before applying algorithms like k-means, Autoregressive Moving Average (ARMA) models, or similar techniques that have been universally used [41,42]. Enhanced variants, such as k-means based on Dynamic Time Warping (DTW) and k-shape based on Shape-Based Distance (SBD), have also demonstrated significant efficacy in handling temporal misalignments [28,29,43]. In contrast, soft clustering offers greater flexibility in handling segments of unequal lengths. Methods based on Latent Dirichlet Allocation (LDA) and its extensions (e.g., GMM-LDA, GW-LDA, and mLDA) can directly capture latent structures from data distributions without rigid feature unification. These approaches are particularly well-suited for the continuous nature of time-series data [27,30,44,45,46] and are increasingly favored for their adaptability and robustness in modeling complex driving behaviors. Currently, deep clustering has established itself as a cutting-edge paradigm and a mainstream solution for high-dimensional data and document clustering tasks. Its core advantage lies in constructing a unified end-to-end framework that achieves joint optimization of feature representation learning and cluster assignment. Under this synergistic mechanism, feature extraction provides a superior semantic space for clustering, while the clustering objective, in turn, guides the shaping of the feature space, with both aspects mutually reinforcing each other. Particularly when dealing with high-dimensional complex data, deep clustering can precisely capture the data’s latent geometric structure and deep semantic information, thereby significantly enhancing the accuracy and robustness of clustering results [47,48].

Research on inferring driving behavior primitives through segmentation and clustering algorithms has garnered significant attention. Despite the advancements in these techniques, significant challenges remain in accurately capturing the nuanced structure of driving behaviors. Current frameworks often treat multi-dimensional inputs as independent streams or oversimplify the temporal evolution within segments, leading to a loss of critical semantic information. Specifically, two fundamental limitations hinder the precise inference of driving behavior primitives:

(1) Neglect of variable correlations in segmentation. Multi-dimensional segmentation methods frequently overlook the intricate correlations and distinctions among input variables. This omission often results in inaccurate change points. For instance, the standard BMASS method commonly suffers from under-segmentation because it fails to fully leverage the dependencies between lateral and longitudinal dynamics.
(2) Loss of temporal dynamics in clustering. Extended Latent Dirichlet Allocation (LDA) methods, while effective for topic modeling, often fail to capture the essential time dependency characteristic of driving behaviors. Since the evolution of actions over time is a defining characteristic of driving maneuvers, ignoring this feature compromises the semantic quality and interpretability of the clustering results.

To address these limitations and accurately infer driving behavior primitives, this paper proposes a two-stage unsupervised framework that combines enhanced H-BMASS and INT-LDA. The H-BMASS structure is designed to segment multi-dimensional driving behavior by explicitly leveraging the correlations and differences between feature variables. By decoupling lateral and longitudinal behaviors and utilizing their information hierarchically, H-BMASS accurately identifies change points in both dimensions, thereby significantly mitigating under-segmentation issues. Within the identified segments, rich contextual information is incorporated by integrating numerical values and trend characteristics into a unified symbolic representation. Subsequently, the INT-LDA method clusters these segments. This approach yields clustering results that better align with the actual driving context, thereby improving the understanding of driver behavioral characteristics and dynamic decision-making processes.

## 2. Data Collection and Feature Variable Selection

Driving data were collected from 17 participants (10 males, 7 females; age: 28–50 years, M = 29.8, SD = 2.7; driving experience: 0–12 years, M = 7.6, SD = 3.3) using an RADS 8-degree-of-freedom panoramic driving simulator. Participants were recruited from the general public. To ensure adequate driving familiarity and behavioral stability, inclusion criteria required a valid driver’s license and a minimum driving frequency of one hour per day over the past three months. Data from one female participant were excluded from the analysis due to a technical failure during the experiment.

The simulated track featured an out-and-back route spanning a total of 43 km, integrating mixed straight and curved sections with realistic traffic and signage. A sparse traffic flow was configured within the virtual environment to simulate the influence of surrounding vehicles on the ego-vehicle during naturalistic driving, alongside randomly deployed trigger-based traffic events. In total, 68 variables were recorded, with 19 specific to vehicle dynamics (e.g., longitudinal/lateral acceleration, yaw and roll rates, sideslip angle, steering angle, and pedal inputs). Gaussian-weighted smoothing was applied to process all collected data.

Selecting feature variables that effectively represent driving behavior is crucial, as utilizing all 19 variables directly for behavior segmentation would reduce computational efficiency and compromise accuracy. For longitudinal driving behavior, speed and longitudinal acceleration were chosen as representative indicators. Regarding lateral driving behavior, lateral acceleration indicates the rate of speed change in the lateral direction, while both yaw angle and yaw rate reflect vehicle steering characteristics. When selecting between yaw angle and yaw rate to combine with lateral acceleration as lateral behavior indicators, it is essential to ensure low correlation with lateral acceleration to enhance feature information utilization efficiency.

Pearson and distance correlation coefficients were calculated for the 17 drivers. For the correlation between yaw angle and lateral acceleration, all values were found to be below 0.2, confirming the absence of significant correlation and thus no information redundancy. In contrast, the correlation between yaw rate and lateral acceleration exceeded 0.7 due to vehicle dynamics relationships. Using the yaw angle to characterize lateral driving behavior not only effectively reduces this correlation but also captures the cumulative change characteristics of vehicle heading through its integration effect. This creates better information complementarity with lateral acceleration, enabling a more comprehensive characterization of lateral driving behavior. Therefore, yaw angle and lateral acceleration were selected as the representative variables for lateral driving behavior.

**Figure 1:**
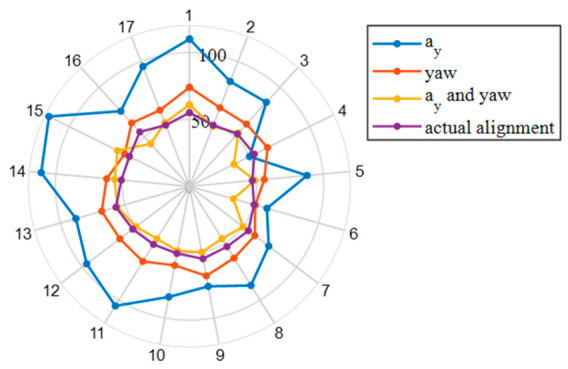
Comparison of trajectory segmentation results using different signals via a rule-based method against actual road alignments. The radial axes indicate the number of segments, while the circular scale represents driver IDs. The purple line depicts the actual road alignment division. The blue, red, and yellow lines correspond to trajectory segmentation results using lateral acceleration, yaw angle, and their combination, respectively.

A rule-based segmentation method utilizing lateral acceleration, yaw angle, and their combination was applied to the driving trajectories. Comparison with the actual road alignment (Figure 1) revealed that the segments derived from the combined feature set most accurately reflected the true alignment. Consequently, both yaw angle and lateral acceleration were adopted as the feature variables representing steering behavior.

## 3. Driving Behavior Segmentation by H-BMASS

### 3.1. The Hierarchical BMASS

Driving behavior primitives are defined as segments in which the vehicle’s state or its rate of change remains consistent. Reasonably partitioning continuous driving behavior into independent segments is critical for analysis.

**Figure 2:**
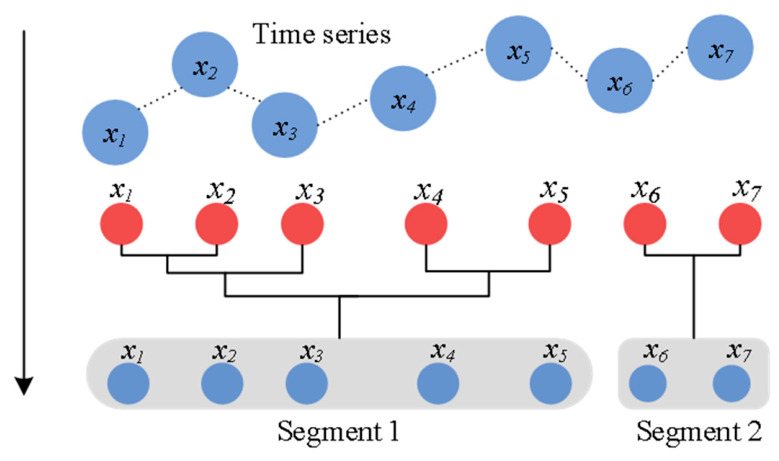
Schematic diagram for series segmentation by BMASS.

The Bayesian model-based agglomerative sequence segmentation (BMASS) algorithm is a robust data-driven method widely used in driving behavior segmentation [27]. It operates as a bottom-up, agglomerative sequence segmentation algorithm that identifies optimal split points to partition the sequence into meaningful segments, as illustrated in Figure 2. Each segment is modeled as a multivariate linear regression, with time serving as the independent variable, as shown in Equation (1). The algorithm employs Bayesian methods to compute the marginal likelihood for each potential split point and evaluates segmentation quality by comparing marginal likelihoods across different partitioning schemes. Starting from the finest initial state, where each data point represents a separate segment, the algorithm iteratively merges adjacent segments. For each merge, the marginal likelihood of the resulting segment is recalculated, and the merge that maximizes the marginal likelihood is selected. This iterative process continues until a predefined stopping criterion is met. By balancing model complexity with data fit, the algorithm yields optimal segmentation results [27,38].

(1) $$y_{i}=A_{0}+At+Q^{\frac{1}{2}}\omega_{i}$$

where $y_{i}$ is the *i*-th variable (there are four variables used in this research for driving behavior segmentation); *t* is time; $\omega_{i}$ Gaussian white noise for the *i*-th variable; $A_{0}$ is the intercept term; *A* is the slope; *Q* is the standard deviation of process noise.

**Figure 3:**
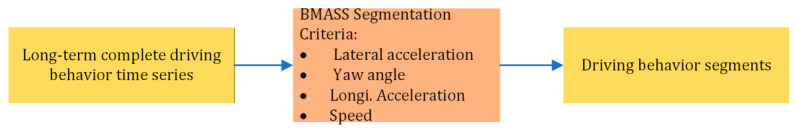
The conventional multivariate time sequence segmentation by BMASS.

Driving behavior is defined by four key variables: longitudinal velocity and acceleration, lateral acceleration, and yaw angle. While the BMASS algorithm is inherently designed for multivariate sequence segmentation, previous studies typically input these variables directly, as depicted in Figure 3.

In multivariate time-series segmentation, the BMASS algorithm identifies change points based on the joint fitting error of all variables. However, if residual magnitudes vary significantly across variables, the algorithm tends to prioritize minimizing error for variables with larger residuals, potentially overlooking those with smaller residuals. Consequently, key transition points for the latter variables may be missed, resulting in under-segmentation. Among the four variables, longitudinal velocity and acceleration typically have much larger numerical magnitudes compared to yaw angle and lateral acceleration. Additionally, these variables show significant disparities in value ranges and standard deviations. These dimensional and scale inconsistencies compromise the effectiveness of the sequence segmentation.

Additionally, the BMASS algorithm implicitly assumes that all four variables are driven solely by time. However, their underlying physical mechanisms differ significantly: longitudinal velocity and acceleration are primarily governed by throttle and brake inputs, whereas yaw angle and lateral acceleration are mainly influenced by steering wheel angle. Beyond temporal effects, these variables are simultaneously subject to two distinct operational factors. Consequently, modeling them purely as linear functions of time fails to capture their dependence on these critical control inputs. A more accurate relationship between the variables and their influence factors should be represented, as shown in Equation (2). This fundamental limitation accounts for the undesired results produced by conventional BMASS segmentation of driving behavior sequences.

(2) $$y_{i}=A_{0}+At+B\times S\left(t\right)+C\times P\left(t\right)+Q^{\frac{1}{2}}\omega_{i}$$

where $S\left(t\right)$ captures the time-dependent intrinsic driving factors of longitudinal driving behavior, primarily governed by the operations of the throttle and brake pedals; $P\left(t\right)$ captures the time-dependent intrinsic driving factor of lateral behavior, mainly governed by the operations of the steering wheel; *B* and *C* are correspondence slopes.

**Figure 4:**
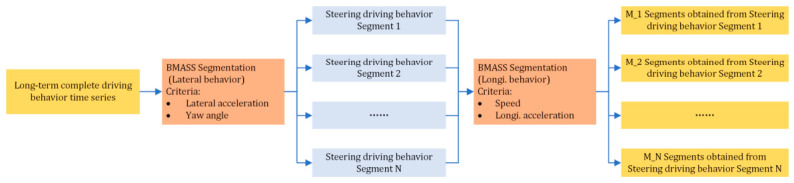
The multivariate time sequence segmentation by H-BMASS.

To achieve segment boundaries that more accurately reflect reality, the hierarchical BMASS (H-BMASS) is proposed, as shown in Figure 4. Addressing the intrinsic differences in factors influencing driving behavior, this approach applies a decoupling process to multi-dimensional data to eliminate cross-influence from other independent variables. Following decoupling, both longitudinal and lateral driving behavior characterization variables are independently modeled as functions of time, as shown in Equation (3). Hence, lateral and longitudinal driving behaviors can be segmented hierarchically. First, behavior segments are identified based on a single dimension at the upper level. Then, information from the other dimension is incorporated to refine these initial segments. By effectively leveraging both lateral and longitudinal features, this method enhances the accuracy and robustness of the segmentation.

(3) $$\left\{\begin{array}{c} y\text{\_}{Lat}_{i}=A_{01}+A_{Lat}\times t+Q_{Lat}^{\frac{1}{2}}\omega_{i}\;\\ y\text{\_}{Longi}_{j}=A_{02}+A_{Longi}\times t+Q_{Longi}^{\frac{1}{2}}\omega_{j}\; \end{array}\right.$$

where ${y\text{\_}Lat}_{i}$ and ${y\text{\_}Longi}_{j}$ are the *i*-th lateral and the *j*-th longitudinal driving behavior representation variables, respectively; $A_{01}$ and $A_{02}$ are intercept terms for lateral and longitudinal driving behaviors, respectively; $A_{Lat}$ and $A_{Longi}$ are their corresponding slopes.

By employing hierarchical segmentation, the computational overhead is significantly reduced by decomposing the original 4-dimensional driving behavior segmentation into two sequential 2-dimensional processes.

Furthermore, a segment-wise processing strategy is implemented during the first level of segmentation (based on lateral driving behavior variables). Instead of processing the entire time-series data for each driver at once, the data are sliced into 2-min windows for BMASS processing. To eliminate boundary effects and segmentation inaccuracies caused by artificial truncation, an overlapping mechanism is adopted, where the tail end of the preceding segment is included in the subsequent 2 min window for re-segmentation.

Through this approach, the segmentation of long sequences is transformed into the processing of multiple shorter sequences. Consequently, the proposed H-BMASS framework, combined with the sliding window strategy, successfully controlled computational costs while maintaining high segmentation accuracy.

### 3.2. Hyperparameter Settings for Behavior Segmentation

In the application of the BMASS algorithm for driving behavior segmentation, two critical hyperparameters are configured: the prior odds of merging, λ, and the prior observation noise variance, Σ.

The prior odds of merging, λ, serve to regulate the granularity of the segmentation. This parameter represents the ratio of the prior probability that adjacent time points belong to the same behavior pattern to the probability that they belong to different patterns before observing the data. The algorithm decides whether to introduce a changepoint by comparing the Bayes Factor (BF) with the reciprocal of the prior odds. A changepoint is identified only when the strength of evidence provided by the data, log (BF), exceeds the threshold −log(λ). To ensure the physical interpretability of the segmentation results, we optimized this parameter by incorporating the physical characteristics of lane-changing maneuvers. Statistical analysis indicates that the duration of the three phases of a lane change (preparation, execution, and adjustment) typically centers around 2 s, providing a prior constraint on the time scale of the primitives. Given the data acquisition frequency of 60 Hz in this study, we conducted sensitivity tests by setting λ within the range of 90 to 150 (with a step size of 10). The results demonstrated negligible differences in segmentation outcomes within this range. Based on this analysis, λ was set to 100.

The prior observation noise variance, Σ, quantifies the algorithm’s expectation of the magnitude of random fluctuations within a segment, essentially defining the boundary between “signal” and “noise”. Within the Bayesian inference framework, Σ directly influences the calculation of the marginal likelihood. A larger Σ diminishes the contribution of data discrepancies to the Bayes Factor, thereby suppressing spurious changepoint detection caused by high-frequency noise. Conversely, a smaller Σ enhances the algorithm’s sensitivity to minor mean shifts. In this study, multivariate driving data (where the first layer of segmentation relies on yaw angle and lateral acceleration and the second on longitudinal velocity and acceleration) are used as criteria for segmentation, and Σ is configured as a diagonal matrix. The diagonal values are derived from the sample variance of a 30 s straight-line driving segment ($\left[\begin{array}{cc} 1 & 0.2\\ 0.2 & 1 \end{array}\right]$ in this research). Since the four variables were preprocessed using Z-score normalization, this adaptive configuration ensures that the algorithm fairly evaluates evidence of behavioral transitions across different dimensions based on actual measurement precision, thereby avoiding detection bias caused by differences in units or scales. If obtaining the empirical noise covariance matrix proves impractical, simply setting Σ to an identity matrix is a highly viable alternative.

### 3.3. Behavior Segmentation Results

**Figure 5:**
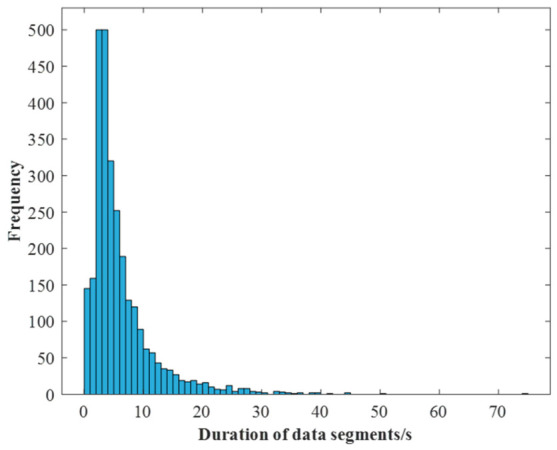
Duration distribution of the obtained 2830 behavior segments.

Initially, lateral acceleration and yaw angle are used as input features to identify steering-related segments, yielding 840 such segments. Subsequently, each of these is further partitioned using speed and longitudinal acceleration to capture longitudinal dynamics, resulting in a total of 2830 fine-grained behavioral segments. The distribution of segment duration is shown in Figure 5.

As shown in Figure 5, the duration distribution exhibits a long tail, indicating significant variability in segment lengths [27,31,32,38]. While most segments fall within the 2–10 s range (average = 6.14 s), a small minority exhibit extreme durations: segments shorter than 1 s or longer than 40 s are observed, with the shortest segment lasting 0.18 s and the longest reaching 74.57 s.

### 3.4. Post-Processing on the Behavior Segments

Segments with excessively long or short durations may exhibit under- or over-segmentation issues. To ensure optimal segmentation quality, the 2830 segments undergo post-processing. Under-segmented segments are further divided, while over-segmented segments are merged with adjacent segments. This process is guided by empirical observation combined with a detailed statistical analysis of the segmented data.

Among the segments, five exceed 40 s in duration, which is significantly longer than that of 95% of the primitives. Visual inspection confirms these correspond to prolonged uniform acceleration during straight-line driving, where the vehicle prepares for subsequent maneuvers. This validates the segmentation rationale.

A total of 145 segments have durations less than 1 s [27,31,32,38]. Through empirical observation, it can be inferred that 68 of them are over-segmented and need to be merged with adjacent segments. The optimal merging boundaries for these segments were determined using the Z-scale to quantify the distances within adjacent segments [5]. To avoid the impact of dimensional units on the results, the data are standardized in advance. Z-scale is carried out using

(4) $$Z=\sum_{u=1}^{N}\sum_{v=1}^{V}\sum_{j=1}^{Nu}\frac{\left|x_{uvj}-\bar{x_{uv}}\right|}{s_{uv}}$$

where $x_{uvj}$ is the *j*-th observation of variable *v* in segment *u*; $\bar{x_{uv}}$ is the average of segment *u* for variable *v*; $s_{uv}$ is the standard deviation of segment *u* for variable *v*; *N* is the number of driving behavior segments; *V* is the number of variables (*V* = 4 in this paper); and *Nu* is the number of observations in segment *u*.

After the post-processing of over-segmented segments merging with adjacent ones, a total of 2746 segments are obtained.

## 4. Segment Clustering by INT-LDA

### 4.1. The INT-LDA for Segment Clustering

Although the sequence segmentation results in a large number of behavior segments, these segments are limited in types and represent the basic driving patterns performed by drivers in response to the environment, known as driving behavior primitives. Therefore, clustering is necessary to group these segments into behavior primitives.

Driving behavior is inherently a continuous modal signal. In practice, however, it is captured by sensors at a fixed sampling frequency and represented as a discrete numerical time series. Consequently, a driving behavior segment corresponds to a finite-length sequence of numerical observations. By discretizing the continuous values into predefined categories, the segment can be transformed into a symbolic sequence analogous to a text document. This representation enables the application of Latent Dirichlet Allocation (LDA) for clustering behavior segments.

LDA is a bag-of-words generative probabilistic model based on the “document–topic–word” paradigm. Its core assumption is that each document is a random mixture of latent topics, and each topic is characterized by a fixed probability distribution over words [49]. To apply LDA to driving behavior segments, the continuous numerical data must first be converted into discrete symbolic representations. Existing studies commonly adopt binning strategies to map raw data points into predefined intervals according to their empirical numerical distribution [27,30,43,44,45].

However, a fundamental limitation of LDA is its disregard for word order: it treats documents as unordered collections of words, thereby ignoring temporal dependencies within sequences. As a result, standard LDA fails to capture the dynamic evolution and sequential patterns inherent in driving behavior segments. This omission can lead to unstable clustering of behavior primitives and, more critically, may produce results that do not faithfully reflect the underlying driving dynamics.

**Figure 6:**
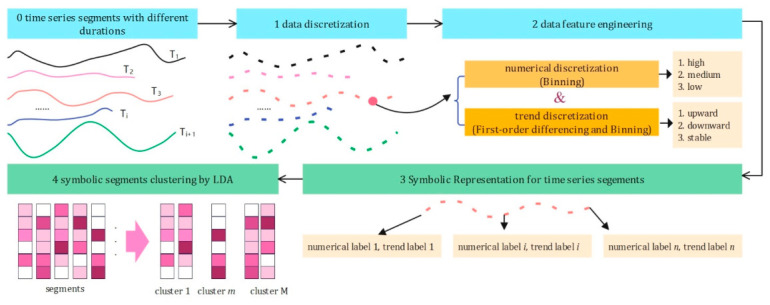
The framework of INT-LDA clustering on behavior segments.

To address this limitation, an enhanced framework termed Integrating Numerical and Trend Discretization LDA (INT-LDA) is proposed, as illustrated in Figure 6. INT-LDA jointly encodes both the magnitude (via numerical discretization) and the local temporal trend (e.g., increasing, decreasing, or steady) of the time-series data into enriched symbolic tokens, thereby preserving essential temporal characteristics while remaining compatible with the LDA modeling framework.

### 4.2. INT Symbolization

During the discretization of driving behavior data, data points are mapped onto a finite set of driving state symbols based on two dimensions: the magnitude of the variable itself and its temporal trend. The discretized representations of numerical values and dynamic trends are combined to transform each data point into a driving state symbol with clear semantic meaning.

#### 4.2.1. Numerical Discretization

Numerical discretization is applied independently to four key driving variables: vehicle speed, longitudinal acceleration, lateral acceleration, and yaw angle. For each variable, binning is performed using empirically informed thresholds derived from prior studies and the statistical distribution of the collected driving behavior data [1,50].

Vehicle speed is partitioned into three discrete levels: low, medium, and high speed. The bin boundaries are defined by the 20th and 90th percentiles of the observed speed distribution, corresponding to 11 m/s (39.6 km/h) and 20 m/s (72 km/h), respectively.

Longitudinal acceleration is discretized into five semantic categories: rapid deceleration, gradual deceleration, constant speed, gradual acceleration, and rapid acceleration. The transition points between these categories are determined by the 30th, 45th, 60th, and 70th percentiles of the longitudinal acceleration distribution, yielding boundary values of −0.18 m/s^2^, −0.05 m/s^2^, 0.07 m/s^2^, and 0.18 m/s^2^, respectively.

Lateral driving behavior is typically discretized into two semantic categories: straight driving and turning maneuvers. While both lateral acceleration and yaw angle serve as indicators of lateral vehicle dynamics, they exhibit distinct characteristics. The yaw angle, representing the vehicle’s heading within the geodetic coordinate system, is a cumulative variable over time. Consequently, it remains relatively stable during straight-line driving and exhibits significant variation primarily during turning events. Directly grouping data based on the distribution of the yaw angle presents challenges, including an excessive number of groups and poor alignment with actual driving scenarios. To address this, the time derivative of the yaw angle is utilized for data distribution-based grouping. This approach yields results that more effectively identify and distinguish turning behaviors.

**Figure 7:**
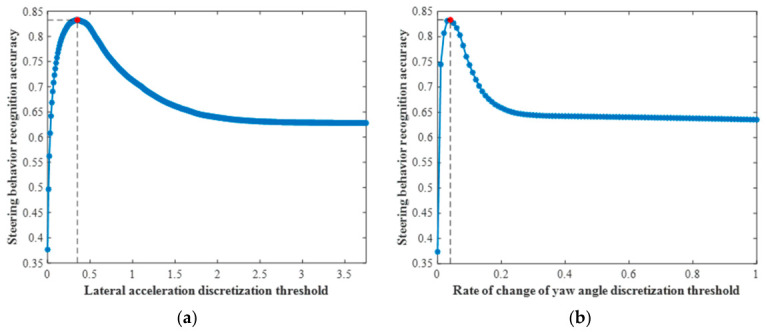
Learning-based determination of interval boundaries for distribution-driven discretization of lateral driving behavior features, guided by ground-truth road alignment labels: (**a**) lateral acceleration; (**b**) rate of change in yaw angle. The red dot denotes the optimal discretization threshold identified through the learning process.

To maximize the accuracy of steering behavior recognition, ground-truth road alignment labels are employed as reference annotations to design a threshold-based road alignment classifier. The optimal binning thresholds for distinguishing between going straight and turning are determined by identifying the threshold values that yield the highest classification performance. As illustrated in Figure 7, the optimal threshold for lateral acceleration is set at 0.35 m/s^2^, achieving an accuracy of 90.10% on straight segments, 71.72% on turning segments, and an overall accuracy of 83.26%. Similarly, the optimal threshold for the rate of change in yaw angle is set at 0.04 rad/s, resulting in 88.80% accuracy for straight sections, 74.08% for turning sections, and an overall accuracy of 83.33%.

Vehicle steering behavior is identified as “turning” if both lateral acceleration and the rate of change in yaw angle simultaneously satisfy the conditions for being within their designated turning intervals.

Table 1 details the numerical discretization scheme, employing binning based on predefined interval boundaries.

#### 4.2.2. Dynamic Trend Discretization

**Figure 8:**
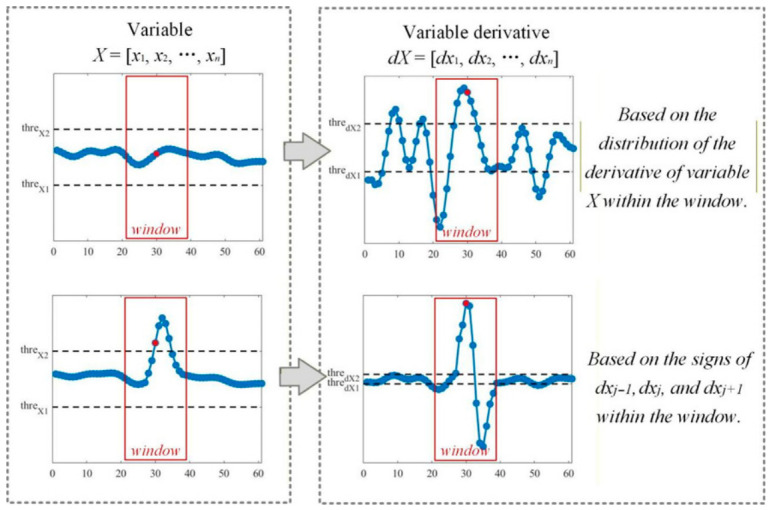
Principles for determining local temporal trends of data points. The red dot denotes the current data point, and the surrounding red box represents the sliding window used to evaluate its local trend. Trend classification rules are derived from the pattern of data fluctuations within this window.

Trend discretization is applied to capture the temporal dynamics of behavior variables and improve the accuracy of segment clustering. Specifically, the change trend of the data point is determined based on the derivatives and fluctuations within a sliding window around it, as shown in Figure 8.

Trend determination is based on the magnitude of a variable’s fluctuation within a sliding window. If the fluctuation remains below a predefined threshold, the trend is inferred from the statistical distribution of its temporal derivatives over the window. Conversely, if the fluctuation exceeds the threshold, the trend is determined by analyzing the sign pattern of the derivatives at the current data point and its immediate temporal neighbors. The resulting trend is classified into one of three categories: upward, downward, or stable.

Suppose data point *x_j_* has the variable sequence and its derivatives within the sliding window are ${SW\text{\_}X}_{j}$ and ${SW\text{\_}dX}_{j}$ separately, and the complete driving behavior sequence is $X=\left[x_{1},\;x_{2},\ldots ,x_{N}\right]$ and its derivative sequence is $dX=\left[{dx}_{1},\;{dx}_{2},\ldots ,{dx}_{N}\right]$.

(1) If $max\left({SW\text{\_}X}_{j}\right)-min\left({SW\text{\_}X}_{j}\right)>\alpha \cdot \left(max\left(X\right)-min\left(X\right)\right)$, the change trend of data point *x_j_* is determined based on the derivative signs at points *x_j−_*_1_, *x_j_* and *x_j+_*_1_. If $|{dx}_{j}|>\alpha \cdot min\left(\left|max\left(dX\right)|,|min\left(dX\right)\right|\right)$, the change trend of *x_j_* (upward or downward) is determined based on the sign of *dx_j_*; if $|{dx}_{j}|\leq \alpha \cdot min\left(\left|max\left(dX\right)|,|min\left(dX\right)\right|\right)$, the change trend of *x_j_* (upward, downward, or stable) is determined based on the sign of *dx_j_*_−1_.
(2) If $max\left({SW\text{\_}X}_{j}\right)-min\left({SW\text{\_}X}_{j}\right)\leq \alpha \cdot \left(max\left(X\right)-min\left(X\right)\right)$, the change trend of data point *x_j_* is determined based on the sign distribution of ${SW\text{\_}dX}_{j}$. In ${SW\text{\_}dX}_{j}$, the number of data points that are within $\left[-\alpha \cdot min\left(\left|max\left(dX\right)|,|min\left(dX\right)\right|\right),\alpha \cdot min\left(\left|max\left(dX\right)|,|min\left(dX\right)\right|\right)\right]$ is denoted as *l*, the number of data points greater than $\alpha \cdot min\left(\left|max\left(dX\right)|,|min\left(dX\right)\right|\right)$ is denoted as *m*, and the number of data points less than $\alpha \cdot min\left(\left|max\left(dX\right)|,|min\left(dX\right)\right|\right)$ is denoted as *n*. If $m>\frac{3}{4}\left(l+m+n\right)$, *x_j_* shows an upward trend; If $n>\frac{3}{4}\left(l+m+n\right)$, *x_j_* shows a downward trend; otherwise, the change trend of *x_j_* is stable.

In this paper, for the purpose of dynamic trend judgement, the sliding window size in dynamic trend judgment is set to 60. Given that the sampling frequency is 60 Hz, this corresponds to a window duration of 1 s. The rationale for this setting is based on the fact that the duration of behavior primitives is typically around 2 s, during which the data trend remains relatively constant. Therefore, a relatively larger window size was chosen as the sliding window scale to ensure the capture of stable trends.

Furthermore, α serves as the fluctuation threshold for data variation within the sliding window. The magnitude of this threshold determines the extent to which data fluctuations are considered negligible noise. Based on the standard deviation and the 90th percentile of the data distribution for each variable, α is set to 0.2 in this study.

**Figure 9:**
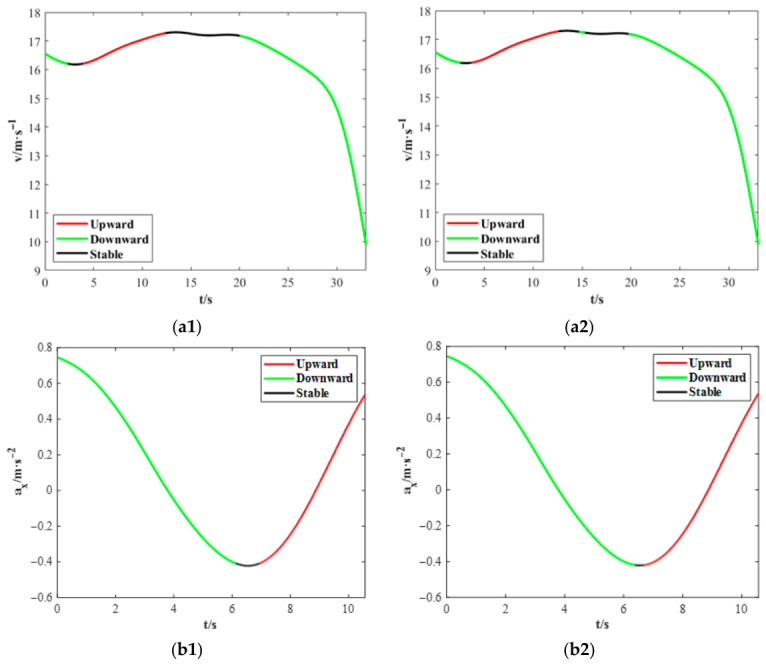
Comparison of temporal trend identification results: (**a1**,**b1**) trends inferred by the proposed method; (**a2**,**b2**) trends derived solely from variable derivatives.

Figure 9 illustrates the trend identification results for longitudinal speed and acceleration in a behavior segment, demonstrating that the proposed method effectively captures the underlying temporal dynamics of the signals.

Figure 9(a2) identifies a downward trend lasting 0.62 s near the 15 s mark, whereas Figure 9(b2) reveals an upward trend persisting for 2.8 s starting at approximately 6.65 s. These transient fluctuations are attributed to sensor noise or minor driving perturbations. Their accurate characterization further demonstrates the effectiveness of the proposed method in jointly leveraging derivative signs and local fluctuation magnitude to robustly determine temporal trends.

### 4.3. Segments’ Symbolic Representation and Clustering

Behavior segments are symbolically encoded by integrating numerical discretization and dynamic trend discretization. Each data point is characterized by four kinematic features: speed, longitudinal acceleration, lateral acceleration, and yaw angle. As detailed in Section 6.2, speed is discretized into three levels (low, medium, and high), and longitudinal acceleration into five semantic levels (rapid deceleration, gradual deceleration, constant speed, gradual acceleration, and rapid acceleration). The combination of lateral acceleration and the rate of change in yaw angle is used to classify lateral behavior into two categories: going straight or turning. Furthermore, each feature variable is annotated with one of three possible local temporal trends: upward, stable, or downward.

Under this joint numerical-trend discretization scheme, the theoretical number of unique symbolic combinations is calculated as 3 × 3 × 5 × 3 × 2 × 3 × 3 = 2430. In the datasets analyzed in this study, 1243 distinct symbols were empirically observed. Segment clustering is subsequently performed using LDA. Three key hyperparameters were configured to optimize the clustering performance. The document-topic prior is set to 0.1, which generally ranges from 0.1 to 1.0. This relatively low value induces sparsity in the document-topic distribution, ensuring that each segment is dominated by a few core topics, thereby enhancing the distinguishability of segments and the clarity of the clustering structure. The topic-word prior is set to 0.02, with a general range from 0.01 to 0.1. A smaller value concentrates the topic-word distribution, which strengthens the semantic coherence and representativeness of topics while preventing over-generalization. Gibbs sampling iteration is set to 2000. This parameter controls the convergence of the Markov Chain Monte Carlo (MCMC) process. Empirical validation confirmed that 2000 iterations are sufficient for convergence metrics, such as log-likelihood, to stabilize, effectively avoiding parameter estimation bias caused by insufficient iterations and ensuring the reliability and reproducibility of the clustering results.

The optimal number of clusters is determined through a dual-criteria evaluation based on perplexity and topic coherence.

Perplexity is a commonly used metric in probabilistic models. It calculates the log-likelihood of the LDA model while considering the total number of samples, and its value reflects the uncertainty of a document belonging to a certain topic, thereby describing the model’s goodness-of-fit to the given samples. Therefore, a lower perplexity value indicates better model performance. The calculation of perplexity is as follows:

(5) $$PP\left(D\right)=exp\left(-\frac{\sum_{i=1}^{I}log\;p\left(w_{i}\right)}{\sum_{i=1}^{I}N_{i}}\right)$$

where $PP\left(D\right)$ is the perplexity value of the entire sample set *D* to be clustered, $log\;p\left(w_{i}\right)$ represents the log-likelihood of all driving data points in the *i*-th primitive sample, $N_{i}$ is the number of data points contained in the *i*-th primitive sample, and *I* is the number of segments included in set *D*.

Topic coherence score is a key metric for evaluating the quality of topic models. It primarily assesses the semantic similarity of words within a topic, determining whether the words under a given topic are semantically “coherent” and “related”. This metric directly reflects the interpretability of the topic. A high coherence score indicates that the words within the topic are highly semantically coherent, making the topic easier for humans to understand and interpret.

**Figure 10:**
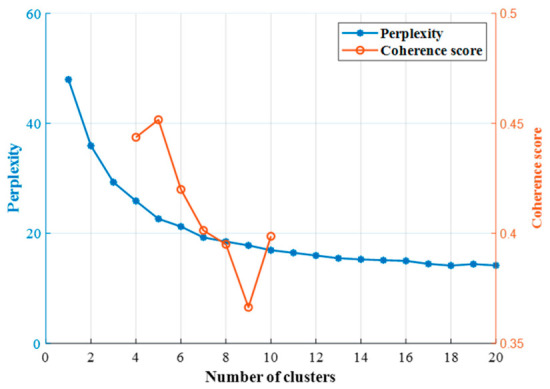
Perplexity and coherence scores under different numbers of clusters.

Each primitive represents a driving pattern, which is a vehicle control mode adopted by a driver in response to environmental changes. Since the number of patterns is not excessively large, we set the number of clusters for the LDA model between 1 and 20. The optimal number of clusters is then determined by comprehensively evaluating both perplexity and the topic coherence score. As shown in Figure 10, perplexity exhibits an elbow-like decline between 4 and 10 clusters, suggesting this range as a candidate set. Within this interval, the coherence score peaks at 5 clusters, which is therefore selected as the optimal configuration. Consequently, five distinct types of behavior primitives are identified in this work.

## 5. Semantic Analysis of Primitives

Semantic analysis is conducted based on the statistical analysis of each cluster (shown in Table 2) to obtain their physical meanings.

**Figure 11:**
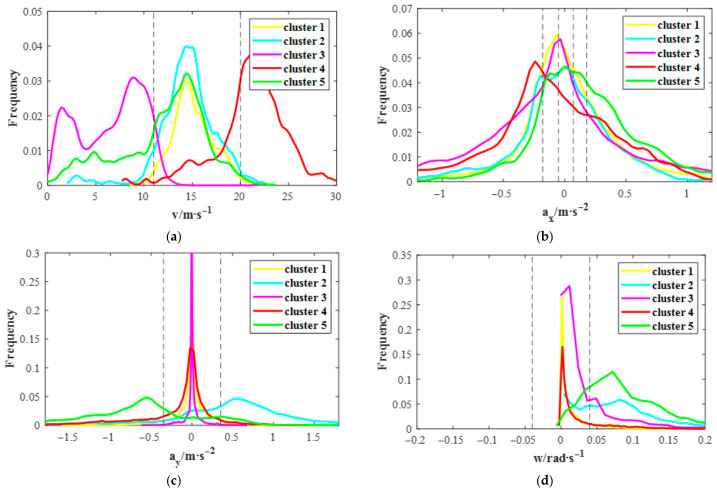
Probability density distributions of five behavior primitive types: (**a**) speed, (**b**) longitudinal acceleration, (**c**) lateral acceleration, and (**d**) rate of change in yaw angle. The vertical dash lines indicate the distribution-based data discretization thresholds.

Figure 11 illustrates the data distribution of speed, longitudinal acceleration, lateral acceleration, and rate of change in yaw angle for each cluster.

**Figure 12:**
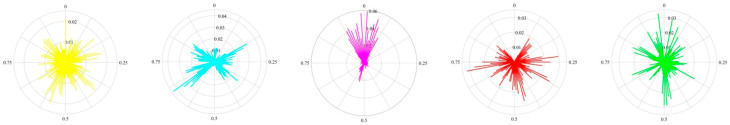
The relative positions and frequencies of primitives appearing in driving trips. From left to right: Cluster 1, Cluster 2, Cluster 3, Cluster 4, and Cluster 5.

The relative temporal positions and occurrence frequencies of each primitive category within driving trips are further analyzed, as illustrated in Figure 12. In this figure, the polar angle represents the relative temporal position, while the polar radius indicates the frequency of occurrence at the corresponding time position.

Clusters 1 and 2 share medium-speed profiles with near-zero mean longitudinal acceleration and low variance, reflecting gradual speed changes. They diverge, however, in their lateral dynamics. Cluster 1, characterized by tightly concentrated steering variables indicative of straight driving, appears throughout all phases of the driving trip. In contrast, Cluster 2, with its predominantly positive lateral acceleration and dispersed rate of change in yaw angle suggesting left turns, is primarily observed during the middle segments of the trip. Consequently, these are defined as “Medium-Speed Straight” and “Medium-Speed Left Turn”, respectively, both characterized by gradual speed variation.

Cluster 3 represents low-speed straight driving with a focus on gradual deceleration, evidenced by low longitudinal metrics and negligible lateral activity. This primitive is frequently observed during the vehicle start-stop phases of driving trips. In contrast, Cluster 4 corresponds to high-speed cruising with subtle speed adjustments. Temporal analysis of the experimental data reveals that primitives belonging to Cluster 4 predominantly occur during the middle stages of the trials. In these instances, drivers executed slight braking maneuvers primarily in response to the dynamics of the surrounding traffic flow or to accommodate scripted trigger events, rather than performing aggressive evasive actions.

Cluster 5 is distinguished by medium speeds and high mean longitudinal acceleration, indicative of rapid acceleration, and appears throughout all phases of the driving trip. Its large magnitude and variance in lateral variables, combined with predominantly negative lateral acceleration, characterize it as a “Medium-Speed Right Turn”.

## 6. Discussion

### 6.1. The Evaluation of H-BMASS Driving Behavior Segmentation

(1) Comparison of Segmentation Results using BMASS and H-BMASS

To evaluate the performance of the proposed H-BMASS method, a comparative analysis against the conventional BMASS method is conducted using the same driving behavior data.

**Figure 13:**
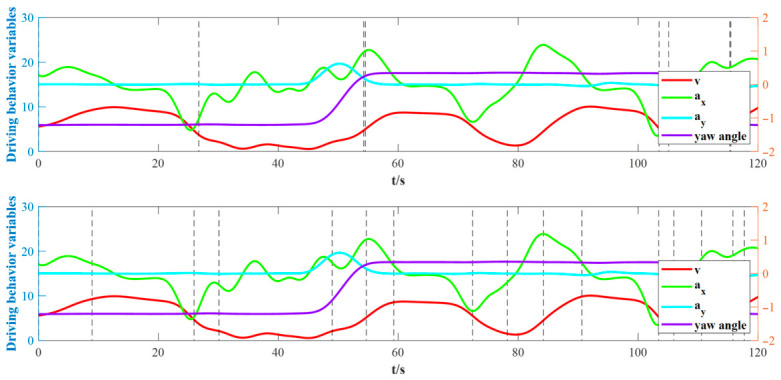
Driving behavior sequence segmentation by conventional BMASS (**up**) and H-BMASS (**bottom**). The vertical dash lines mark the boundaries between segments.

A visual comparison of a representative segment is provided in Figure 13. As shown, the conventional BMASS method fails to detect several critical behavioral transitions. Specifically, it misses changes in turning behavior at 50.3 s and 112.6 s, as well as shifts in longitudinal behavior at 73.1 s and 87.4 s. Furthermore, it leads to excessive fragmentation, such as the split at 57.3 s, which creates segments with impractically short time intervals.

This observation is further supported by the results from segmenting the data of 16 drivers. The conventional BMASS method produced significantly fewer segments (1253) compared to the H-BMASS method (2746). This discrepancy indicates that BMASS tends to under-segment the data by inconsistently overlooking change points, sometimes in the longitudinal behavior and at other times in the lateral behavior.

(2) Validation of Segmentation Effectiveness Based on the Chow Test

Since the extraction of driving behavior primitives is an unsupervised, data-driven approach, ground-truth labels are unavailable to directly verify the segmentation accuracy. Instead, the method proposed in this research relies on a fundamental premise that a primitive is defined as the minimal, physically meaningful segment of driving behavior, within which the variation trends of the driving variables remain essentially constant. Consequently, a single primitive can be approximated by a multivariate linear regression model. Based on this assumption, the validity of the segmentation is evaluated through a structural comparison of adjacent segments. The H-BMASS algorithm yields a valid segmentation only when adjacent segments exhibit statistically distinct linear regression models. These models are fitted using Maximum A Posteriori (MAP) estimation, while the structural differences are rigorously validated via the Chow test to confirm behavioral shifts at the segmentation boundaries.

**Figure 14:**
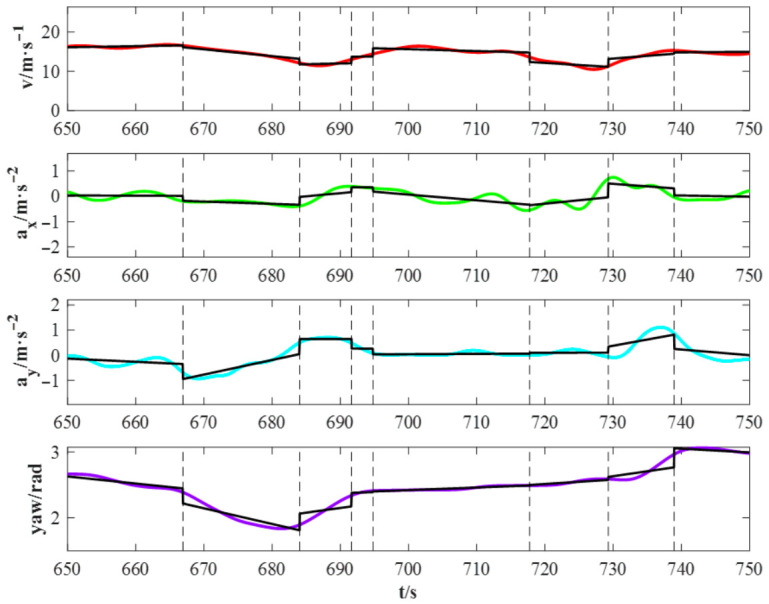
Example of linear regression models for adjacent segments derived via MAP estimation from a representative driving behavior sequence. The vertical dash lines indicate the segment boundaries inferred by H-BMASS, while the solid black lines represent the linear trends within each segment.

Operating within a Bayesian framework, MAP estimation treats parameters as random variables, integrating prior knowledge with observed data through likelihood functions. Within each segment, MAP is employed to estimate the linear regression coefficients of feature variables with respect to time. Figure 14 illustrates the resulting linear regression models for adjacent segments derived via this MAP-based approach.

Two-tailed tests were employed with a significance level of 0.05. The Chow test was applied to 2746 pairs of adjacent segments, revealing statistically significant structural changes at every segmentation point identified by H-BMASS. These results robustly validate the effectiveness of the proposed behavior segmentation method.

### 6.2. The Evaluation of INT-LDA Segment Clustering

For comparative analysis, we also performed clustering based exclusively on numerical discretization (referred to as the LDA model), alongside the proposed INT-LDA model. In both setups, the number of clusters was set to K = 5.

While perplexity is commonly used to evaluate topic models, it is not suitable for a direct comparison between the LDA and INT-LDA models in this study. This is because the two models employ different discretization methods for the driving behavior data, rendering their perplexity values incomparable.

Therefore, entropy is employed to evaluate the quality of the clustering results, which can be obtained using Equation (6). As a widely used metric in soft clustering, entropy measures the uncertainty of the category to which samples belong. In the context of topic distribution, lower entropy signifies a purer distribution and reduced uncertainty, indicating superior clustering performance.

(6) $$S\left(D\right)=\sum_{i=1}^{I}\left(-\sum_{k=1}^{K}p\left(k\right)log\;p\left(k\right)\right)$$

where $S\left(D\right)$ denotes the entropy of the set of data segments to be clustered; *p* represents the topic distribution of each document.

The experimental results demonstrate a significant improvement with the proposed model. The standard LDA model yielded an entropy of 1017.440, whereas the INT-LDA model achieved 762.909. This represents a 25.02% reduction in entropy, confirming that INT-LDA produces more distinct and reliable clustering results.

**Figure 15:**
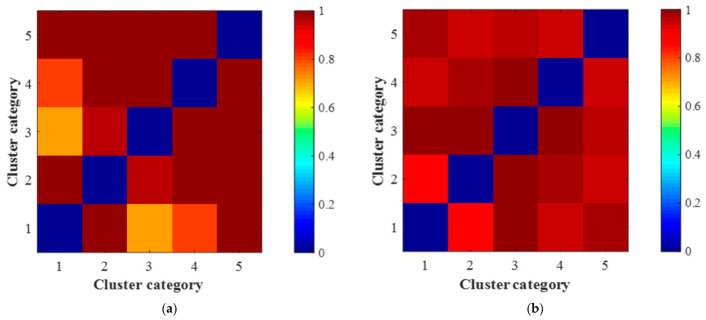
Comparison of topic-word distributions between clusters generated by LDA and INT-LDA: (**a**) LDA and (**b**) INT-LDA. Blue indicates minimal discrepancy between the corresponding clusters, whereas red signifies substantial divergence.

Furthermore, Jensen–Shannon (JS) divergence was employed to evaluate the distributional distinctiveness between clusters. Higher JS divergence indicates greater separation between clusters, implying better model discriminability. Figure 15 visualizes the topic-word distributions for both models. While the average JS divergence improved modestly from 0.759 (LDA) to 0.770 (INT-LDA), which is only a 1.45% increase, a deeper inspection of Figure 15 reveals critical structural improvements. Specifically, under the LDA model, the JS divergence between Cluster 1 and Cluster 3, as well as between Cluster 1 and Cluster 4, is notably low, indicating poor distinguishability among these groups. In contrast, the INT-LDA model effectively resolves this issue, exhibiting clear separation across all cluster pairs and eliminating the ambiguity observed in the baseline LDA approach.

While entropy and JS divergence are commonly used to measure the structural quality of unsupervised clustering (i.e., pursuing intra-cluster compactness and inter-cluster separation), high intra-cluster consistency and large inter-cluster differences are not sufficient to directly confirm that the clustering results align with objective reality. To rigorously validate the effectiveness of INT-LDA, this study further examines the consistency of linear regression coefficients of feature variables among data segments within the same primitive category. Specifically, the MAP method described in Section 6.1 is employed to estimate the linear regression coefficients of feature variables over time for each driving behavior data segment. Subsequently, the correlation of these regression coefficients for all data segments belonging to the same primitive category is calculated. If the clustering results are accurate, data segments within the same category should exhibit a high degree of consistency in the trends of their physical features (i.e., high correlation).

Experimental results demonstrate that for data segments clustered into the same primitive by INT-LDA, the correlations of the regression coefficients for the four feature variables are all above 0.84, whereas the correlation for LDA clustering results is only 0.69. This finding provides strong evidence that INT-LDA is superior to the traditional LDA model not only in terms of statistical distribution separation but also in the intrinsic consistency of actual physical features.

### 6.3. Research Limitations and Further Work

This paper employs a data-driven, unsupervised approach to study driving behavior. Consequently, the results are inevitably influenced by factors such as data scale, subject demographics, and the data acquisition environment. To extract robust driving primitives under low-noise conditions, this study intentionally controlled subject variables (limiting participants to experienced, regular drivers) and environmental variables (using standardized simulator scenarios). This strategy effectively mitigated anomalous behavior categories caused by driving inexperience or extreme stress, ensuring the clarity and interpretability of the primitive clustering results.

However, real-world traffic environments are highly complex, and traffic participants cover a broad spectrum of demographic characteristics. To comprehensively explore the types and characteristics of driving primitives and to use them as fundamental units for in-depth research on driving styles and decision-making strategies, it is necessary to discuss the limitations of this study and outline future research directions.

(1) Sample size and demographic coverage

Constrained by the rental costs of high-fidelity, high-degree-of-freedom driving simulators and the expenses associated with recruiting subjects for social study, this study adopted a controlled experimental design to maximize internal validity. During recruitment, subject age was strictly limited to the 28–50 range, and all participants were required to meet a “recent high-frequency driving” criterion (driving at least one hour daily over the past three months). This strategy aimed to minimize interference noise arising from age-related physiological and cognitive changes (e.g., reaction speed, physical stamina), ensuring that observed behavioral differences stemmed primarily from scenario complexity and individual strategic choices rather than physiological age differences. This allowed the study to validate the effectiveness of the proposed primitive extraction method and perform semantic parsing of primitives more purely and robustly within a limited sample size.

Nevertheless, drivers with different demographic backgrounds and driving experiences may exhibit distinct primitive types. Young adults versus the elderly, and novice versus expert drivers, construct underlying driving modes that differ significantly due to variations in physiological characteristics, psychological stress tolerance, and accumulated experience. Investigating these group-level differences in driving patterns represents a highly valuable direction for future research.

(2) Discrepancies between simulation and real-world environments

Significant differences exist between driving simulator environments and real-world traffic conditions, which may introduce potential biases in the definition and distribution of behavioral primitives. Although the experiments attempted to replicate real scenarios by setting sparse traffic flows and pre-scripted events, the simulator environment still falls far short of real roads regarding the diversity, complexity, and dynamic game-theoretic nature of vehicle-to-vehicle interactions.

In terms of interaction frequency and intensity, driving on real urban branch roads involves extremely high-frequency and high-intensity interactions between the ego-vehicle and neighboring vehicles. These interactions are characterized by non-linear game-theoretic features (e.g., yielding, competing for right-of-way, cooperative lane changing). Furthermore, real-world traffic often involves concurrent multi-target interactions, where drivers must simultaneously process complex relationships with multiple neighboring vehicles. The high-dimensional concurrent decision-making pressure is difficult to fully reproduce in a simulator.

Compared to the diverse interactions in real traffic, the monotonous and limited interactions in the simulator may lead to two specific biases in the primitive analysis results:

✓ Underestimation of primitive richness. Even when extracting primitives based solely on ego-vehicle kinematic indicators, the subtle manipulation patterns generated by complex game-theoretic situations in the real world would likely result in a significantly larger number of primitive categories than in the current simulator environment. The current set of primitives may omit specific driving modes that are only triggered under strong interaction or high-risk game-theoretic conditions.
✓ Absence of interactive primitives. Current research focuses primarily on ego-vehicle kinematic features, failing to fully capture the core dimension of interaction. In real environments, the essence of many driving behaviors is an interactive strategy (e.g., “aggressive cut-in” or “defensive following”), which cannot be fully defined by the ego-vehicle’s driving state alone. Future definitions of primitives urgently need to incorporate vehicle-to-vehicle interaction states (e.g., relative distance, relative velocity, time to collision) as supplementary bases to extract “interactive primitives” with richer semantic information.

(3) Data supplementation and extraction of interactive primitives

This research serves as an exploration of methodological paradigms. Supported by this methodology, a new round of data collection planning will be initiated.

✓ Expanding subject scope: Simplified driving simulators will be used to broaden recruitment, focusing initially on young drivers (younger age and relatively less experience) to fill the gaps in the current dataset regarding low-age/low-experience dimensions. Relying on driving data from these diverse groups, primitives will be extracted, and differences across age groups and experience levels will be systematically compared. Based on these preliminary comparative results, larger-scale validation experiments with greater demographic diversity will be further designed to comprehensively evaluate the generalization capability of the proposed method.
✓ Incorporating interactive primitives: Driving behavior data from real traffic environments will be supplemented. On the basis of recording ego-vehicle operating states, interaction states between the ego-vehicle and neighboring vehicles will be simultaneously collected. Based on the definition of effective interaction intervals, the extraction and representation of interactive primitives will be explored. An interactive primitive will be defined as a data segment where the interaction state between the ego-vehicle and neighboring vehicles remains constant. Driving behavior primitives based on ego-vehicle states reflect the vehicle operation modes of drivers facing diverse environments and are considered “result-oriented primitives”. In contrast, interactive primitives reveal how neighboring vehicles influence the ego-vehicle and engage in games and are considered “cause-oriented primitives”. By leveraging both interactive and driving behavior primitives, we will be able to explore a richer array of driving behavior research topics.

## 7. Conclusions

Behavior primitives are fundamental to achieving a deep understanding of driving behaviors. This study focuses on inferring these primitives from multi-dimensional data collected via a driving simulator to partition continuous driving streams into the smallest physically meaningful units. By leveraging the H-BMASS and INT-LDA algorithms, sequential behaviors are segmented into independent intervals and clustered into distinct, interpretable groups, ultimately identifying five primitive clusters. These clusters comprise three straight-driving patterns and two turning patterns, reflecting diverse driver responses to varying traffic environments.

Cluster 1 is characteristic of medium-speed straight driving with near-zero mean longitudinal acceleration and low variance, reflecting gradual speed changes. This cluster appears throughout all phases of the driving trip. Cluster 3 represents low-speed straight driving with a focus on gradual deceleration. This primitive is frequently observed during the vehicle start-stop phases of driving trips. Cluster 4 corresponds to high-speed cruising with subtle speed adjustments. Temporal analysis reveals that this cluster predominantly occurs during the middle stages of the trials, where drivers execute slight braking maneuvers primarily in response to surrounding traffic flow dynamics or scripted trigger events, rather than performing aggressive evasive actions.

Clusters 2 and 5 correspond to left and right turns, respectively. It is worth noting that while the simulated environment successfully captured the turning patterns, the variability in speed profiles and fluctuations within these turning primitives was somewhat constrained compared to real-world complexity.

Each primitive represents a specific driving pattern. When executing continuous driving tasks, drivers select and combine corresponding primitives for operation in response to changes in the external environment. Therefore, by taking behavior primitives as the basic unit and analyzing the distribution characteristics and transition patterns of primitives over long-duration driving tasks, the time-varying and dynamic driving styles of drivers can be characterized. This not only facilitates an accurate understanding of whether drivers maintain consistency in driving styles or risk preferences across different environments but also enables in-depth analysis of drivers’ decision-making characteristics by exploring the transition patterns and frequencies of primitives. This analysis would provide valuable metrics for characterizing driving habits, thereby enhancing risk prediction and driver warning systems.

If the operational states of the ego-vehicle and the interaction states between the ego-vehicle and surrounding vehicles are synchronously collected, the primitive extraction method proposed in this paper can be applied to obtain both the ego-vehicle’s driving behavior primitives and the interaction primitives between the ego-vehicle and surrounding vehicles. The ego-vehicle’s driving behavior primitives serve as result-oriented primitives, while the interaction primitives between the ego-vehicle and surrounding vehicles function as cause-oriented primitives. Critical insights into driver decision-making processes and interaction strategies with other traffic participants are provided by these interaction primitives. Through the synchronous analysis of both types of primitives, the behavioral decision-making strategies of drivers can be explored. Such understanding is pivotal for validating Large Language Models (LLMs) and Vision–Language Architectures (VLAs) in autonomous driving contexts. Furthermore, these primitives can be systematically combined to construct diverse and high-fidelity test scenarios for autonomous vehicles.

## Figures and Tables

**Table 1:** Binning configuration for numerical discretization of the four driving behavior variables.

Variables	Intervals	Symbols	Code Representation
Speed (m/s)	*v* ≤ 11 m/s	Low Speed	*a*
	11 m/s < *v* ≤ 20 m/s	Medium Speed	*b*
	*v* > 20 m/s	High Speed	*c*
Longitudinal Acceleration (m/s^2^)	*a_x_* ≤ −0.18 m/s^2^	Rapid Deceleration	*d*
	−0.18 m/s^2^ < *a_x_* ≤ −0.05 m/s^2^	Gradual Deceleration	*e*
	−0.05 m/s^2^ < *a_x_* ≤ 0.07 m/s^2^	Constant Speed	*f*
	0.07 m/s^2^ < *a_x_* ≤ 0.18 m/s^2^	Gradual Acceleration	*g*
	*a_x_* > 0.18 m/s^2^	Rapid Acceleration	*h*
Lateral Acceleration (m/s^2^)	−0.35 m/s^2^ ≤ *a_y_* ≤ 0.35 m/s^2^	Going straight	*s*
	*a_y_* < −0.35 m/s^2^, *a_y_* > 0.35 m/s^2^	Turning	*t*
Yaw Angle (rad)	Range of Change per Second ≤ 0.04 rad	Going straight	*s*
	Range of Change per Second > 0.04 rad	Turning	*t*

**Table 2:** Statistical features of behavior primitive clusters.

Variables	Statistical Features	Cluster Category				
		Cluster 1	Cluster 2	Cluster 3	Cluster 4	Cluster 5
Speed (m/s)	mean	15.09	14.47	6.62	21.12	11.95
	std	2.18	3.11	3.49	3.72	4.58
Longitudinal Acceleration (m/s^2^)	mean	0.00	−0.03	−0.12	−0.11	0.15
	std	0.44	0.44	0.63	0.55	0.45
Lateral Acceleration (m/s^2^)	mean	−0.01	0.44	0.00	−0.23	−0.62
	std	0.27	0.75	0.12	0.58	0.73
Rate of Change in Yaw Angle (rad/s)	mean	0.02	0.11	0.03	0.03	0.14
	std	0.03	0.19	0.13	0.04	0.21

## Data Availability

The datasets generated during and/or analyzed during the current study are available from the corresponding author upon reasonable request.
